# Winter behavior of bats and the progression of white‐nose syndrome in the southeastern United States

**DOI:** 10.1002/ece3.2772

**Published:** 2017-02-05

**Authors:** Riley F. Bernard, Gary F. McCracken

**Affiliations:** ^1^ Department of Ecosystem Science and ManagementPennsylvania State UniversityUniversity ParkPAUSA; ^2^ Department of Ecology and Evolutionary BiologyUniversity of TennesseeKnoxvilleTNUSA

**Keywords:** acoustic activity, bats, echolocation, white‐nose syndrome, winter captures

## Abstract

Understanding the winter behavior of bats in temperate North America can provide insight into how bats react to perturbations caused by natural disturbances such as weather, human‐induced disturbances, or the introduction of disease. This study measured the activity patterns of bats outside of their hibernaculum and asked how this winter activity varied by time, temperature, bat species, body condition, and WNS status. Over the course of three winters (2011–2013), we collected acoustic data and captured bats outside of five hibernacula in Tennessee, United States. During this time, Pseudogymnoascus destructans, the causative agent of white‐nose syndrome, became established in hibernacula throughout the region, allowing us to track disease‐related changes in the winter behavior of ten bat species. We determined that bats in the southeastern United States were active during winter regardless of disease. We recorded activity outside of hibernacula at temperatures as low as −13°C. Although bat activity was best determined by a combination of variables, the strongest factor was mean daily temperature (*R* ^2^ = .2879, *F*_1,1450_ = 586.2, *p* < .0001). Bats that left the hibernacula earlier in evening had lower body condition than those that left 2–4 hr after sunset (*F*_7,932_ = 7.225, *p* < .0001, Tukey HSD, *p* < .05). The number of daytime emergences from hibernacula, as determined via acoustic detection, increased the longer a site was P. destructans positive (*F*_3,17 808_ = 124.48, *p* < .0001, Tukey HSD, *p* < .05). Through the use of passive acoustic monitoring and monthly captures, we determined that winter activity was driven by both ambient temperature and the presence of *P. destructans*.

## 1 Introduction

Winter activity is documented for several species of North American bats that use roost sites with variable microclimates such as foliage, bark, or man‐made structures (Boyles, Dunbar, and Whitaker, 2006). However, information on winter behavior of North American cave‐roosting bats is based largely on studies at latitudes above 40°N, or in desert regions of the southwestern United States (US; Boyles *et al*. 2006). Previous studies of winter activity of bats in Ontario, New England, Indiana, and Missouri found that individuals arousing during winter months mostly flew within the hibernaculum, and rarely left the cave (Griffin 1945; Whitaker and Rissler, 1992; Boyles *et al*. 2006). These findings reinforce the assumption that bats enter hibernation sites in the fall and are not observed on the landscape until mid‐spring. Prior to the white‐nose syndrome (WNS) disease epizootic, it was suggested that bats leave hibernacula in winter infrequently, and do so to switch roosts or in search of water or food (Boyles *et al*. 2006). However, as WNS became established in the northeast, behaviors such as daytime and cold‐weather flight during winter became indicative of infection. To date, no studies have looked at the winter behavior of bats in the lower latitudes of the southeastern USA, where warmer temperatures and available insects may allow for sustained activity through winter.

The importance of understanding the winter behavior of bats in the USA has become better appreciated due to WNS. First identified in February 2006 in a cave in New York, this disease has spread across eastern North America as far west as Missouri, south into Mississippi, and north into Canada, and was recently found further west in Washington state. To date, WNS has killed over 5 million bats of seven hibernating species (USFWS, 2014). Mortality as high as 90%–100% has been documented at some northeastern hibernacula, leading to possible regional extinction of several once‐common species (Frick *et al*. 2010; Langwig *et al*. 2012). The highest rates of morbidity and mortality at sites in the northeast have been documented following the second and third winters after initial visual detection of the fungus (Titchenell 2012; Knudsen, Dixon & Amelon 2013).

White‐nose syndrome is caused by the psychrophilic (cold‐tolerant) fungus, *Pseudogymnoascus destructans* Minnis & Lindner (Lorch *et al*. 2011; Minnis & Lindner 2013), a non‐native pathogen from Eurasia (Leopardi, Blake & Puechmaille 2015; Hoyt *et al*. 2016). *Pseudogymnoascus destructans* invades the muzzle, wings, and tail membrane of bats during torpor when their immune systems are likely suppressed (Moore *et al*. 2011; Field *et al*. 2015). Invasion of the wing tissue by *P. destructans* can cause dehydration, electrolyte imbalance, and increased arousal frequency leading to critical loss of fat stores needed to survive hibernation (Cryan *et al*. 2010; Reeder *et al*. 2012; Warnecke *et al*. 2012, 2013, Verant *et al*. 2014, 2016). Changes in winter behavior associated with WNS have been documented, with bats roosting in exposed regions of cave entrances or leaving hibernacula during the day, and flying during cold winter nights (Turner, Reeder & Coleman 2011; Foley *et al*. 2011; Carr, Bernard & Stiver 2014). To date, seven bat species have been confirmed with the disease via histopathology, with five additional species confirmed with *P. destructans* DNA on their epidermis (Meteyer et al. 2009; USFWS, 2014; Bernard *et al*. 2015).

Tennessee is among several southeastern states that contain hibernacula used by threatened and endangered species, including *Myotis sodalis* Miller & Allen, *M. septentrionalis* Trouessart, and *M. grisescens* Howell. The fungus was first detected in Tennessee during winter 2009–2010 at the largest *M. sodalis* hibernacula in the state (Samoray 2011; Carr *et al*. 2014). By winter 2015–2016, 54% of the counties in Tennessee (*n* = 52/95) were deemed WNS positive, with several high‐priority *M. sodalis* hibernacula experiencing declines in bat populations (Campbell 2016). *Myotis septentrionalis* appears to be one of the hardest hit species in Tennessee, having experienced declines varying from 69% to 98.5% at known hibernacula (TWRA, unpublished data). Although declines due to WNS have not been confirmed in *M. grisescens*, they were thought to be vulnerable to the disease due to their propensity for forming high‐density clusters during hibernation (U.S. Fish and Wildlife Service, 2009). *Myotis grisescens* are known to hibernate in eight caves in Alabama, Arkansas, Kentucky, Missouri, and Tennessee (USFWS, 1982, 1997). In vitro growth curves suggest that *P. destructans* may reproduce more quickly in cave environments that maintain more moderate temperatures of 10–15°C in winter (Verant *et al*. 2012), which could result in increased virulence in southern hibernacula.

Our study measured the activity patterns of bats outside of caves during winter and investigated how this activity varied by time, temperature, bat species, body condition, and WNS status. To address these questions, we looked at the following hypotheses: (1) Bats will remain active throughout winter in the southeast due to warmer ambient temperatures than in the northeast and (2) Length of WNS infection at hibernacula will affect the body condition and winter activity of cave‐roosting bats. Passive acoustic monitoring and bat captures during winter outside of caves allowed us to address these questions without adding additional stress to bats within hibernacula.

## 2 Materials and Methods

### 2.1 Study area

**Figure 1:**
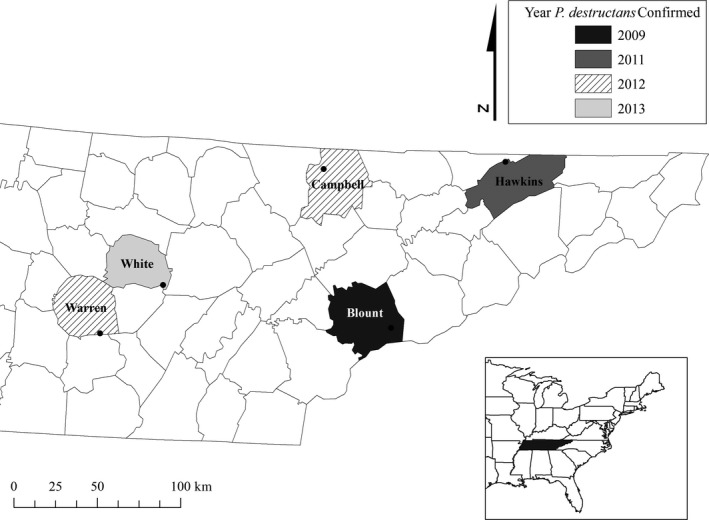
Cave locations within county boundaries in Tennessee, United States. Color coding of counties corresponds to the year *Pseudogymnoascus destructans* was confirmed using either real‐time PCR (Muller et al. 2013) or histopathology (Meteyer et al. 2009)

Bat activity was monitored at three hibernacula in Middle and East Tennessee during January – May 2012 (year 1), and five hibernacula during October – May 2012–2013 (year 2) and 2013–2014 (year 3; Figure 1). Blount cave is the largest known *M. sodalis* hibernaculum in Tennessee, with an estimated 9,500 individuals in winter 2012–2013 (Flock 2013). Hawkins and Warren caves are two of the largest *M. grisescens* hibernacula in the state and also contain small populations of *M. sodalis*. Recent hibernacula surveys estimated 150,000 and 500,000 endangered *M. grisescens* at Hawkins and Warren caves, respectively. Campbell and White caves contain *Corynorhinus rafinesquii* Lesson*, Eptesicus fuscus* Beauvois*, M. leibii* Audubon and Bachman*, M. lucifugus* Le Conte*, M. septentrionalis,* and *M. sodalis* (Holliday 2012). White cave had approximately 700 *M. grisescens* during winter 2012–2013. Four bat species hibernated in Blount cave, and approximately six species of bat (*n* = <10,000 individuals) were counted during hibernacula surveys at Campbell cave (Samoray, 2011; Flock, 2013).

### 2.2 Acoustic activity

Ultrasonic bat detectors were deployed near the entrance of each hibernaculum. Acoustic data were recorded in zero‐crossing mode onto 4 Gb compact flash (CF) cards (year 1) and 32 Gb secure digital (SD) cards (years 2 and 3). Detectors were placed approximately 25–40 m from each cave entrance. During year 1, three Anabat II detectors with CF ZCAIM units (Zero‐Crossing Analysis Interface Module; Titley Electronics, Ballina, New South Wales, Australia) were used at Blount, Campbell, and Warren caves. Microphones were housed in waterproof “bat hats” with reflector plates (Arnett, Hayes & Huso 2006) and secured on PVC pipes approximately 3 m above ground level. All electronic equipments (Anabat II detector, ZCAIM and two 12‐volt batteries in parallel) were enclosed in large utility boxes (Duluth Trading Company, Belleville, WI, USA) located at the base of the PVC pipe.

Due to technical difficulties with aging Anabat II and ZCAIM units, SM2Bat+ detectors (Wildlife Acoustics, Maynard, Massachusetts, USA) programed to record data in zero‐crossing, were used in years 2 and 3. Detectors were redeployed at Blount, Campbell, and Warren caves and added to White and Hawkins caves. SM2Bat+ detectors were secured to a 30.5 × 20 cm piece of plywood and attached to a 5‐cm‐diameter PVC pipe using U‐bolts. The SMX‐US microphone was attached to a 1‐m cord and fastened at a 45° angle to the top of the PVC pipe. The SM2Bat+ detectors were powered by a 6‐volt external battery enclosed in utility boxes at the base of the entire unit.

Because WNS‐affected bats in the northeast are often active during daytime, bat acoustic activity and temperature were recorded 24 hr/day. Data cards and batteries were replaced monthly. Acoustic data were collected from 4 January to 30 May 2012 (Blount, Campbell & Warren caves; *n* = 361 acoustic days), 4 October 2012 – 7 May 2013 (Blount, Campbell, Hawkins, Warren & White caves; *n* = 1,004 acoustic days), and at these same caves 2 October 2013 – 13 May 2014 (*n* = 1,001 acoustic days). During the three winters, acoustic monitoring was conducted on 538 days at Blount cave, 498 days at Campbell cave, 384 days at Hawkins cave, 543 days at Warren cave, and 403 days at White cave. Acoustic monitoring for the last 2 years of the study was from the end of fall swarming through spring emergence. Temperature was recorded at each site at 15‐min intervals using HOBO U‐series data loggers with an accuracy of ± 0.21°C (Onset Computer Corporation, Bourne, MA, USA). Each temperature meter was 2.5 m above the ground and 10–15 m from the acoustic detector.

### 2.3 Bat captures

During years 2 and 3, bats were captured near the entrance of each cave once a month using 6‐, 9‐, and 12‐meter mist nets (Avinet, Dryden, New York, USA; 75/2 mesh size, 2.6 m high, 4 shelves, black polyester for bats). Nets were deployed for a total of 6,899.1 net hr/m^2^ over both winters. Each site had designated equipment to prevent the spread of *P. destructans* among sites. Mist nets were deployed 30 min before civil sunset and remained open for 5 hr, until 30 bats were captured, or temperatures dropped below freezing (0°C). Bats were held individually in paper bags and placed in a large insulated box with four hand warmers (HotHands^®^, Dalton, Georgia, USA) for 30–60 min prior to processing. Each captured bat was identified to species, and reproductive condition (scrotal, pregnant, lactating, postlactating, nonreproductive), right forearm length (mm), weight (g), and wing damage index (WDI) score were recorded. Guano was collected when possible. Wing damage was classified from 0 to 3, where WDI score = 0 indicates no obvious scaring or discoloration on the membranes, WDI score = 1 denotes light damage covering less than 50% of the membranes, WDI score = 2 indicates moderate damage greater than 50% of the wing membrane covered in scar tissue, and WDI score = 3 signifies heavy damage with deteriorated wing membranes and necrotic tissue (Reichard & Kunz 2009). In year 3, bat wings were examined using ultraviolet (UV) light to check for lesions indicative of WNS. Under UV light, damage caused by *P. destructans* infiltration will fluoresce yellow orange (UV positive), allowing for confirmation of WNS infection without submitting the bat for histological confirmation (Turner *et al*. 2014). Ultraviolet examination was not used in years 1 and 2 of our study because the method had not yet been substantiated. To asses overall health of each bat, body condition indices (BCI) were calculated by dividing weight by forearm length (Speakman & Racey 1986). All species, except Lasiurans and *Corynorhinus*, were banded with either a 2.4‐mm or 2.9‐mm numbered forearm band and released on site.

All capture and handling techniques were approved by the University of Tennessee Institute of Animal Care and Use Committee (protocol 2026‐0514) and are consistent with the guidelines issued by the American Society of Mammalogists (Sikes, 2016).

### 2.4 Data analysis

Acoustic activity was quantified as the number of files or “bat passes” recorded per 24‐hr period. A “bat pass” was defined as a file containing a search‐phase echolocation sequence of ≥2 echolocation pulses (Gannon, Sherwin & Haymond 2003). This metric is an index of overall activity and does not correlate to the numbers of individual bats actively flying outside of the cave (Kunz *et al*. 2007; Schwab & Mabee 2014). All call files were analyzed in AnalookW (version 3.9c, Titley Scientific) using antinoise filters. Filters were used to eliminate files that contained calls with less than two pulses, calls with short duration (<4 ms) or low frequency noise (i.e., wind, insect noise, or rain). Once all noise files were removed, each file was manually vetted to determine whether the remaining call files fit the “bat pass” criteria. Because we were attempting to quantify bat activity at each site regardless of species, and due to the difficulties in distinguishing the closely similar calls of several Myotine species (Barclay 1999; Britzke *et al*. 2011), species identification of each call was not attempted for this study.

Generalized linear mixed models (GLMM) with site as a random effect were calculated using the package lme4 (version 1.1‐11) in R (version 3.2.4; R Core Development Team, 2016). Negative binomial GLMMs were employed to account for overdispersion and repeated sampling. Differences in bat acoustic activity among years were tested using daily and hourly “bat pass” totals. Fixed effects included year, sampling season (winters 2011–2012, 2012–2013, and 2013–2014), month, hibernation period (early—[October 1 – December 15], mid—[December 16 – February 15], late—[February 16 – April 30]), time of day (day or night), hour (0:00 to 23:00 hr), mean daily temperature (24‐hr period), temperature at time of emergence (dusk), relative humidity, moon illumination, and year since *P. destructans* confirmation at each cave. Year since *P. destructans* confirmation was based on the detection of fungal DNA on epidermal swab samples or histology of voucher specimens (USGS National Wildlife Health Center 2013). Nonparametric Kruskal–Wallis and Mann–Whitney–Wilcoxon tests were used to examine differences between species in frequency of capture and BCI among month, site, across seasons, and year since *P. destructans* confirmation. Post hoc pairwise comparisons using Tukey and Kramer (Nemenyi) tests were used to determine differences among factors using the package PMCMR (version 4.1).

## 3 Results

### 3.1 Acoustic activity

Useable acoustic data were collected on 88.4% or 2,091 of 2,366 detector nights. Loss of data resulted from malfunctioning Anabat/ZCAIM units, SM2Bat+ external AC adaptor failure and a microphone cord failure. The recordings contained a total of 566,000 bat passes with bat activity recorded on 1,695 of the 2,091 recording days. Mean activity at sites varied considerably (Table 1), presumably due to differences in species compositions and population sizes at each location. Daily bat activity was best predicted by the model that included the interaction between month and temperature at emergence with the additive effects of year since *P. destructans* was confirmed and moon illumination (Table 2). Hourly bat activity was best described by the model that included the interaction of time of day (day or night classification) with mean hourly temperature, and year since *P. destructans* was confirmed (Table 2).

**Table 1:** Mean number of bat calls per period ± *SE* and mean temperature (°C) ± *SE* per site from January through April 2012 (^a^indicates truncated season) and October through April 2012–2013 and 2013–2014. Mean number (#) of bat calls and temperature from sunrise to sunset are reported as “Day” and those from sunset to sunrise as “Night”. Hawkins and White caves were added to the sampling regime in winters 2012–2013 and 2013–2014. Mean activity at Blount cave during winter 2013–2014 is high due high call activity in October 2013 (b). The temperature meter at White cave malfunctioned in winter 2012–2013

Cave	Time of day	Winter survey year					
		2011–2012^a^		2012–2013		2013–2014	
		Mean # bat calls	Mean temp. °C	Mean # Bat Calls	Mean temp. °C	Mean # bat calls	Mean temp. °C
Blount	Day	3.55 ± 0.34	15.03 ± 0.18	5.34 ± 0.29	8.95 ± 0.18	0.15 ± 0.07^b^	11.67 ± 0.19
	Night	21.74 ± 0.84	9.42 ± 0.17	6.69 ± 0.27	4.41 ± 0.16	8.64 ± 0.96^b^	6.48 ± 0.16
Campbell	Day	19.88 ± 3.16	No temp meter	0.15 ± 0.02	8.49 ± 0.16	1.63 ± 0.11	9.34 ± 0.21
	Night	131.56 ± 8.33	No temp meter	11.22 ± 0.75	6.25 ± 0.12	17.67 ± 0.71	5.47 ± 0.15
Hawkins	Day			0.50 ± 0.21	11.02 ± 0.21	0.50 ± 0.09	13.44 ± 0.26
	Night			7.54 ± 0.80	4.08 ± 0.13	29.95 ± 1.37	4.83 ± 0.19
Warren	Day	7.39 ± 0.88	No temp meter	0.63 ± 0.10	10.65 ± 0.24	1.38 ± 0.25	5.57 ± 0.26
	Night	87.13 ± 2.73	No temp meter	24.34 ± 0.96	7.33 ± 0.19	18.35 ± 1.15	5.23 ± 0.21
White	Day			0.25 ± 0.06	Meter broke	0.10 ± 0.01	5.81 ± 0.17
	Night			7.78 ± 0.44	Meter broke	2.48 ± 0.18	5.20 ± 0.14

**Table 2:** Top five models identifying variables that best explain daily and hourly acoustic activities of bats during winter in the southeastern United States. Daily acoustic activity is the average number of bat calls per 24‐hr period, whereas hourly acoustic activity is the average number of bat calls per hour. Akaike information criterion (AIC) values identify the best‐fit model. Degrees of freedom (*df*), change in AIC (ΔAIC), and weight of evidence (wi) for the models relating bat activity to abiotic variables are shown for all sites and seasons in all years. Models receiving substantial empirical support (ΔAIC ≤ 2.0) are in bold face type

	Explanatory variables in model	*df*	ΔAIC	wi
Daily acoustic activity	Month * Emerg. Temp. + Yr Pd Confirmed + Moon Illumination	**3**	**0.0**	**1.0**
	Month + Emerg. Temp. + Yr Pd Confirmed + Moon Illumination	4	19.1	0.0
	Month * Emerg. Temp. + Moon Illumination	2	161.9	0.0
	Month + Emerg. Temp. + Moon Illumination	3	165.7	0.0
	Hibernation Period + Emerg. Temp. + Yr Pd Confirmed + Moon Illumination	4	181.8	0.0
Hourly acoustic activity	Time of Day * Mean Temp. + Yr Pd Confirmed	**2**	**0.0**	**1.0**
	Time of Day * Mean Temp.	1	25.9	0.0
	Time of Day + Mean Temp. + Yr Pd Confirmed	3	995.3	0.0
	Time of Day + Mean Temp.	2	1035.6	0.0
	Hour * Mean Temp. + Yr Pd Confirmed	2	5699.7	0.0

* Indicates interaction between two variables.

(+) indicates additive effects.

Bat calls were recorded on nights where temperatures at emergence were below 0°C on 23 nights at Blount cave, 27 nights at Campbell cave, eight nights at Hawkins cave, three nights at Warren cave, and three nights at White cave. The lowest temperature at which bats were detected was −13°C (*n* = 2 calls), and bats were not detected on nights when the temperature at emergence was below −8°C (*n* = 49 nights). Bat activity was significantly positively correlated with mean daily temperature (*R* ^2^ = .2879, *F*_1,1450_ = 586.2, *p *<* *.0001). The highest mean numbers of nightly calls were recorded during year 1, with a reduction in total calls recorded in subsequent years (Table 1). There was an increase in daytime activity at Blount cave in year 2, 2 years post‐WNS confirmation, followed by a sharp decline in total acoustic activity by the end of year 3. However, at all sites, nighttime acoustic activity always exceeded daytime activity. The highest number of bat calls recorded per day (mean ± *SE*; 1,252.53 ± 39.14 bat calls) was recorded at caves that were *P. destructans* negative during the first year of monitoring (Campbell and Warren caves; chi‐square = 325.72, *df* = 4, *p *<* *.0001). Four caves experienced significant declines in mean nightly bat calls each year following *P. destructans* confirmation (*F*_2, 39 845_ = 1,051.63, *p *<* *.0001, Tukey HSD, *p *<* *.05). However, nightly bat activity at Hawkins cave increased significantly between years 2 and 3 (Wilcoxon = 4010.5, *p *<* *.0001), despite similar mean nightly temperatures for both years (Table 1). The number of daytime emergences from hibernacula increased the longer a site was *P. destructans* positive (*F*_3,17 808_ = 124.48, *p *<* *.0001, Tukey HSD, *p *<* *.05).

### 3.2 Bat captures

A total of 947 individuals of 10 species were captured (Table 3). These included 648 males, 297 females, and two *M. leibii* of unknown sex due to escape. Almost all bats captured (*n* = 936) were cave hibernators; however, eight *Lasiurus borealis* Müller and three *Lasionycteris noctivagans* La Conte were captured; both are migratory species that do not typically use caves as hibernacula. Netting attempts were most successful on mild nights where mean temperatures ranged between 11 and 15°C. *Lasiurus borealis* was captured on nights when average temperatures during the 5 hr following emergence were above 11°C, with all other species captured as low as 8°C and one *M. grisescens* captured on a night where the mean nightly temperature was 0°C. Across all seasons, the most bats were captured in October (*n* = 190) and April (*n* = 222), the first and last months of the hibernation period. More bats were captured during winter 2012–2013 (year 2; *n* = 531) than in winter 2013–2014 (year 3; *n* = 416; chi‐squared = 51.19, *p *<* *.0001). There was a significant decline in the number of individuals captured at a site as the time since confirmation of *P. destructans* increased (chi‐squared = 1387.01, *p *<* *.0001). Most bats captured during the study period (*n* = 871) had a WDI = 0. Sixty‐four individuals (6.75%) scored a WDI = 1, with the remaining individuals (*n* = 10; 1.15%) scoring WDI = 2–3. Of these 10 bats, four were *M. grisescens*, four were *M. septentrionalis*, and two were *P. subflavus* (Reboredo‐Segovia, A. Reboredo Segovia, R. F. Bernard, E. V. Willcox, R. T. Jackson, M. Patton, & G. F. McCracken, unpublished).

**Table 3:** Total bat captures by species in 2012–2013 and 2013–2014. Nets were deployed for a total of 6,899.1 net hr/m^2^ (winter 2012–2013 = 3,182.4 net hr/m^2^, winter 2013–2014 = 3,716.7 net hr/m^2^) at a capture rate of approximately 0.137 bats per net hr per m^2^

Species	Winter 2012/13			Winter 2013/14		
	Females	Males	Total	Females	Males	Total
CORA	1	3	4	2	3	5
EPFU	7	13	20	6	13	19
LABO	1	2	3	2	1	3
LANO	0	0	0	1	2	3
MYGR	31	133	164	54	128	182
MYLE	17	35	52^a^	10	40	51^a^
MYLU	7	5	12	3	16	19
MYSE	39	103	142	24	32	56
MYSO	25	27	52	6	39	45
PESU	33	49	82	8	25	33

Species acronym code: CORA, *Corynorhinus rafinesquii*; EPFU, *Eptesicus fuscus*; LABO, *Lasiurus borealis*; LANO, *Lasionycteris noctivagans*; MYGR, *Myotis grisescens*; MYLE, *Myotis leibii*; MYLU, *Myotis lucifugus*; MYSE, *Myotis septentrionalis*; MYSO, *Myotis sodalis*; PESU, *Perimyotis subflavus*.

a One MYLE from each year escaped prior to collecting any biometric information, including sex.

**Figure 2:**
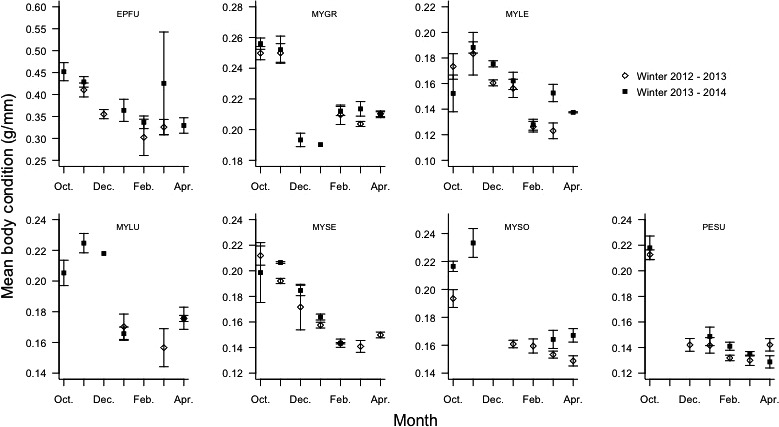
Mean body condition (body mass (g)/forearm length (mm)) of seven bat species captured at all five hibernacula. Individuals captured in winter 2012–2013 (open diamonds) had significantly lower body condition from mid‐ to late hibernation (January through April) when compared to individuals captured during the same period in winter 2013–2014 (black squares). Species acronym code: EPFU,* Eptesicus fuscus*; MYGR,* Myotis grisescens*; MYLE,* Myotis leibii*; MYLU,* Myotis lucifugus*; MYSE,* Myotis septentrionalis*; MYSO,* Myotis sodalis*; PESU,* Perimyotis subflavus*

Across all seasons, body condition (BCI) was highest in October, declined during December – February, and began to increase during March and April, the last two months of hibernation (*F*_6, 312_ = 42.9653, *p *<* *.0001). There were no significant differences in mean BCI between males and females. Mean BCI for all species captured during January through April of year 3 was significantly higher than for the same months in year 2 (*t*_940_ = 7.69, *p *<* *.0001; Figure 2). Bats captured in the first 2 hr after sunset had the lowest BCI on average, with those captured several hours after emergence having higher mean BCI (*F*_7,932_ = 7.225, *p *<* *.0001). Individuals that provided fecal samples had a higher mean BCI than those that did not defecate while in captivity (*t*_940_ = 3.57, *p *=* *.0004). All 290 fecal pellets collected contained recently consumed prey (as described by Dunbar *et al*. 2007; R. F. Bernard, V. A. Brown, E. V. Willcox, & G. F. McCracken, unpublished). There was no significant difference between mean BCI and WDI of bats captured during the study. Body condition varied in relation to how long a site was *P. destructans* positive (*F*_4, 937_ = 12.87, *p *<* *.001; Tukey HSD, *p *<* *.05). Bats captured at caves positive for 2 years had the highest mean BCI (0.222 ± 0.005g/mm, *n* = 107), followed by sites positive for 1 year (0.206 ± 0.003g/mm, *n* = 269), 4 years (0.197 ± 0.005 g/mm, *n* = 51), and 3 years (0.190 ± 0.004 g/mm, *n* = 206). Bats captured at caves that had yet to be confirmed as positive for *P. destructans* exhibited the lowest mean BCI (0.182 ± 0.003 g/mm, *n* = 309). Individuals identified as UV fluorescence‐negative (*n* = 477) had higher mean BCI than UV fluorescence‐positive bats (*n* = 68; *t*_137.8_ = −7.31, *p *<* *.0001).

## 4 Discussion

This is the first study to demonstrate that bats are active throughout winter in the southeastern USA. Activity throughout winter was not restricted to bats in WNS‐infected caves; however, increases in activity associated with low temperatures and diurnal flight were attributed to the disease. Variation in body condition across all species was found throughout a capture session, across seasons, and in relation to how long a hibernaculum was *P. destructans* positive.

### 4.1 Winter activity

Hibernating mammals arouse periodically throughout winter to presumably maintain physiological balance and activate suppressed immune systems (Prendergast *et al*. 2002; Field *et al*. 2015); however, prior to the arrival of WNS, bats in northern latitudes were rarely found leaving the hibernacula (Whitaker and Rissler, 1992; Schwab & Mabee 2014). Czenze *et al*. (2013) noted that *M. lucifugus* in Canada (53°N 99°W) entered a hibernaculum in mid‐September and remained there until mid‐May, showing no signs of emergence. Nevertheless, moderate bat activity has been reported throughout winter in some high‐latitude regions that experience mild oceanic climates (Park, Jones & Ransome 2000; Hope & Jones 2012; Burles *et al*. 2014). Continental and Great Lake effects during winter in the northeast where WNS has caused the most dramatic population declines, generally result in severe weather. We recorded and captured bats that were active during winter in more mild southern climates, and fecal samples from bats captured outside of caves verify that bats were leaving the hibernacula to forage (Bernard *et al*., unpublished). We captured bats representing every species of cave‐dwelling bat known to hibernate in the region, as well as tree and foliage‐roosting species, demonstrating that it is not only the latter that remain active during winter (Boyles *et al*. 2006). Both cave and tree or foliage‐roosting species captured were active throughout winter at a range of temperatures, indicating that bats overwintering in the southeast exhibit different behaviors than northern populations (Boyles *et al*. 2006). This is an important factor for understanding latitudinal differences in WNS susceptibility and mortality (Bernard *et al*., unpublished).

We found evidence for a strong correlation of temperature with winter activity of bats in all 3 years at all study caves. Winter temperatures at each site varied significantly during the study period with the warmest temperatures recorded in year 1. While activity levels were impacted by temperature, they were confounded by disease and differed by species. The apparent effects of WNS are seen in the seasonal activity for the species most affected by the disease (i.e., *M. lucifugus*,* M. septentrionalis*, and *P. subflavus*). However, changes in acoustic activity at caves dominated by *M. grisescens*, which are as of yet, little affected, were linked to temperature, not disease.

In the northeast, peak mortality occurred 2–3 years after the first detection of WNS infections, with populations of *M. lucifugus* and *P. subflavus* experiencing slower rates of population decline 3–4 years post‐WNS detection (Langwig *et al*. 2012). In the southeast however, significant declines were not documented until year 3 of this study, which was nearly 5 years after the first confirmed WNS infections. By the second year of monitoring, bats hibernating at caves with large populations of WNS‐affected species exhibited expected disease‐related behavioral changes. Bats at Blount cave were recorded flying during the day (Carr, Bernard & Stiver 2014), with the number of calls recorded in April of year 2 equivalent to mid‐hibernation levels, indicating reduced activity during spring emergence. In October of year 3, bats at Blount Cave were recorded at similar numbers to the previous year; however, activity dropped off significantly by January, with no bats recorded at the cave after February. Over 80% of the bats captured at Blount cave in March and April of year 3 had visible signs of WNS, including wing damage or ultraviolet (UV) fluorescence (Turner *et al*. 2014) and lower than average body condition. At Campbell cave, the total number of bats captured decreased significantly by the third year of sampling, with an increase in the number of *P. subflavus* found roosting outside of the cave. We did not see the same mortality effects of WNS as seen in the northeast in years 2–3 until 4–5 years after the disease was detected in the southeast. Therefore, hibernating bat populations in the southeast may continue to experience delayed declines caused by WNS due to regional differences in climate‐related life‐history traits such as shorter hibernation periods (Sherwin, Montgomery & Lundy 2013), abbreviated torpor bouts (Brack and Twente, 1985; Twente, Twente, and Brack, 1985; Jonasson & Willis 2012), and foraging opportunities throughout winter (Bernard *et al*., unpublished).

Winter activity and capture rates documented at Hawkins, Warren, and White caves, which are dominated by *M. grisescens*, remained consistent throughout our study to pre‐ or early WNS detection years. We credit the decreases in acoustic activity during the second and third years of sampling to colder temperatures rather than to the effects of WNS. *Myotis grisescens* are the largest bodied Myotine species in eastern North America and hibernate in clusters of 100,000 to over one million individuals (USFWS, 2009). Living in large colonies is expected to promote density‐dependent transmission of the pathogen through increased contact rates (Ryder *et al*. 2007; Langwig *et al*. 2012); however, *M. grisescens* are currently surviving hibernation without any obvious declines due to WNS (Flock 2014; USFWS, 2014). Studies examining load and prevalence of *P. destructans* on hibernating bat populations have found that less than 20% of *M. grisescens* sampled are infected with the fungus (Bernard *et al.,* unpublished; Janicki *et al*. 2015). *Myotis grisescens* are the only eastern *Myotis* species to roost in caves year round (Decher & Choate 1995). They preferentially hibernate at temperatures as low as 1–9°C (Tuttle 1976; Tuttle & Kennedy 2005) and roost in dense clusters of more than 1,800 bats/m^2^ (Gore 1992). Although WNS has been confirmed in *M. grisescens*, we believe their large body size and relaxed energetic constraints for hibernating through shorter winters may prevent *P. destructans* from reproducing at disease‐inducing levels. While these concepts have yet to be investigated, they could provide insight as to why *M. grisescens* populations are so far persisting during the WNS epizootic.

### 4.2 Bat captures and body condition

Over the course of two winter seasons, we captured twice as many males as females flying outside of each hibernaculum. One explanation for sex bias in captures of bats active in winter is the “thrifty female hypothesis” (Jonasson and Willis 2011). This theory suggests that adult females minimize energy loss by relying more on deep torpor during hibernation, whereas adult males have more energy to expend and rely less on torpor. The capture of more active males than females is consistent with this idea. However, we did not detect any difference in body condition between males and females, nor did we find any seasonal patterns in the rate of decline in BCI for males versus females, as would be expected if females were remaining in caves to preserve energy. The “thrifty female hypothesis” may best explain sex ratio bias in bats hibernating in more northern latitudes and may function best where winters are more severe and intermittent foraging is not feasible like it is in the southeast.

An alternative explanation may be due to disproportionate sex ratios of bats within hibernacula (Whitaker & Gummer 2000; Parsons *et al*. 2003). Previous studies suggest sex ratios at winter sites are biased toward males at more northern roosts and become more evenly distributed the farther south a hibernacula is located, with sites in Florida reported to have an equal distribution of males and females (Davis 1959; Tinkle & Milstead 1960; Elder & Gunier 1978). Samanie Loucks and Caire (2007), however, described hibernating colonies of *M. velifer* in Oklahoma (*n* = 42 caves) to be female‐biased throughout winter, suggesting seasonal movements of males and females throughout the hibernation period can change sex ratios frequently. Although we netted at each site once per month throughout winter, a single sex ratio determination for a hibernacula may not accurately reflect the true value for the entire hibernation season. While sample sizes were small, we saw no obvious gender bias in *C. rafinesquii* which uses abbreviated torpor during hibernation and continue to forage throughout winter (Johnson *et al*. 2012), or in *L. borealis* and *L. noctivagans*, which hibernate in more thermally unstable environments (Table 3). These observations suggest that both sexes of these species may be similarly active in winter in foraging or searching for alternative roosts.

## 5 Conclusions

In the southeastern USA, bats are active outside of hibernacula and feed on insects throughout winter regardless of WNS disease status. Activity was found to be strongly correlated with temperature; however, activity during daytime and subfreezing temperatures increased following the confirmation of *P. destructans* at a site, suggesting that vulnerable bat species in the southeast exhibit behaviors similar to those seen at WNS‐infected hibernacula in the northeast. The onset of aberrant behavior and mortality occurred 4–5 years after WNS confirmation, which was substantially later than what has been documented in the North. We found regional differences in the bat faunas affected by WNS. Caves dominated by *M. septentrionalis*,* M. sodalis,* and *P. subflavus* displayed increased aberrant behaviors and reduced activity toward the end of year 3, whereas *M. grisescens* populations showed no such effects. We also identified differences in how bats prepare for winter, with individuals in the southeast entering hibernation with lower mean BCI than bats studied in the northeast. Mild winter climates and available insects in the southeast can allow for shorter hibernation and continued foraging, likely reducing the demands of fat storage prior to winter. The regional differences in bat behavior we identified in this study provide a better understanding of the adaptive variations of bats to climatic perturbations and diseases. These findings are relevant to how bat populations across North America will react to infection by *P. destructans* and can allow for more targeted intervention and disease management.

## Conflict Interest

None declared.
